# Antiobesity Effect of* Astilbe chinensis* Franch. et Savet. Extract through Regulation of Adipogenesis and AMP-Activated Protein Kinase Pathways in 3T3-L1 Adipocyte and High-Fat Diet-Induced C57BL/6N Obese Mice

**DOI:** 10.1155/2018/1347612

**Published:** 2018-12-09

**Authors:** Xian Hua Zhang, Zhiqiang Wang, Bueom-Goo Kang, Seung Hwan Hwang, Jae-Young Lee, Soon Sung Lim, Bo Huang

**Affiliations:** ^1^Department of Food Science and Engineering, Jinzhou Medical University, Jinzhou 121001, China; ^2^College of Public Health, Hebei University, Baoding, 071002, China; ^3^Department of Food Science and Nutrition, Hallym University, Hallymdeahak-gil, Chuncheon 24252, Republic of Korea; ^4^Department of Biochemistry, School of Medicine, Hallym University, Hallymdehak-gil, Chuncheon 24252, Republic of Korea

## Abstract

*Astilbe chinensis *Franch. et Savat. (AC) has been used in traditional medicine for the treatment of chronic bronchitis, arthralgia, and gastralgia. In this study, we investigated the antiobesity effect of AC extract on 3T3-L1 preadipocytes and high-fat-diet-fed C57BL/6N obese mice. We found that AC extracts dramatically decreased the lipid content of 3T3-L1 cells in a concentration-dependent manner without cytotoxicity. The action mechanism of AC extract was demonstrated to be the inhibition of lipid accumulation and dose-dependent decrease in the expression of CCAAT/enhancer-binding protein *α* (C/EBP*α*), peroxisome proliferator-activated receptor-*γ* (PPAR-*γ*), and sterol regulatory element-binding protein 1 (SREBP1). Furthermore, AC extract increased the mitochondrial phosphorylation of AMP-activated protein kinase (AMPK) and acetyl-CoA carboxylase (ACC), mitochondrial biogenesis, and lipolysis-related factors. In amice model of high-fat-diet-induced obesity, the mice administered AC extract experienced significant decrease of 64% in weight gain, 55% in insulin resistance index, 22% in plasma triglycerides (TG), 56% in total cholesterol (TC), and 21% in nonesterified fatty acid (NEFA) levels compared with those in the high-fat diet-fed control mice. Collectively, these results indicated that AC extract exerted antiobesogenic activity through the modulation of the AMPK signaling pathway, inhibition of adipogenesis, decreased lipid content, and reduced adipocyte size.

## 1. Introduction

The increased incidence of obesity, which is associated with the increased intake of calorie-rich food and a sedentary lifestyle, has become a global health problem over the last 40 years. Obesity increases the risk of type 2 diabetes mellitus, hypertension, dyslipidemia, and cardiovascular disease, which all reduce life expectancy [1, 2]. Obesity is major health burden, with annual costs exceeding $100 billion [3, 4]. The World Health Organization has identified obesity as one of the major emerging chronic diseases of the 21st century. Many researchers and companies have therefore undertaken the development of therapies to control obesity. The Food and Drug Administration (FDA) has approved a number of antiobesity agents that act on the central nervous system, such as orlistat, lorcaserin, and phentermine/topiramate (Qsymia) [5]. These drugs can produce adverse effects, including pulmonary hypertension, cardiovascular toxicity, stroke, nonfatal cardiovascular events, and neuropsychiatric issues [6]; therefore, the plant kingdom has emerged as a popular resource for natural effective weight loss drugs that have a broad range of action and minimal or no adverse effects.

*Astilbe chinensis *Franch. et Savat. (AC), a member of the Saxifragaceae family, is a perennial herbaceous plant that grows at an altitude of 390–3600 m in Russia, China, Korea, and Japan [7, 8]. In traditional medicine, its rhizome has been used to treat chronic bronchitis, arthralgia, headache, and gastralgia [9]. Pharmacological experiments have demonstrated the antineoplastic and immunopotentiating activities of extracts from AC [8], such as flavonoids, steroids, and triterpenoids [10]. However, there are no reports documenting the antiobesity effect of AC. In the present study, we evaluated the effects of AC extract on 3T3-L1 preadipocyte differentiation and investigated its underlying effect on body weight gain, lipid droplet accumulation, lipolysis, and gene expression in high-fat-diet-fed obese C57BL/6N mice.

## 2. Materials and Methods

### 2.1. Preparation of AC Extract

The whole plant of AC extract was purchased from Gangwon Medicinal Herbs (Chuncheon, South Korea) and authenticated by Professor-Heung Jun Chi, a medicinal botanist. The dried AC (1.0 kg) was extracted by 30% ethanol-water solution (v/v) (10 liters) at 70°C for 6 hours (h). The extract was filtered using filter paper and concentrated under a vacuum evaporator. The concentrated extracts were freeze-dried by PVTED10R (Ilshinbiobase Co., Ltd., Yangju, Korea).

### 2.2. High Performance Liquid Chromatography (HPLC) Analysis of AC Extract

HPLC equipment was an Agilent technologies 1200 series system and the chromatographic separations were performed using a Gemini column (150 × 4.6 mm i.d., 5 *μ*m particle size; Phenomenex). The mobile phase consisted of solvents A (0.1% aqueous trifluoroacetic acid) and B (acetonitrile). The gradient elution was modified as follows: from 0 to 10 min, 0-20% B; from 10-15 min, 20-20% B; from 15-25 min, 20-25% B; from 25-35 min, 25-30% B; from 35-40 min, 30-30% B; from 40-50 min, 30-100% B. A flow rate was 1 mL/min, and injection volume was 10 *μ*L. UV detected at 280 nm. Determinations for triterpenoids of* Astilbe chinenesis* extracts were performed according to the methods by Moon* et al*. [9].

### 2.3. 3T3-L1 Preadipocyte Differentiation and Oil Red O Staining

The 3T3-L1 preadipocyte cell were obtained from the ATCC, and was grown in DMEM medium containing 10% fetal bovine serum (FBS), 100 U/mL of penicillin, and 100 *μ*g/mL of streptomycin at 37°C under 5% CO_2_. On 2 days after confluence, preadipocytes of 3T3-L1 cell (day 0) were cultured in differentiation medium (DMEM containing 1 *μ*M dexamethasone, 0.5 mM 3-isobutyl-1-methylxanthine, 10 *μ*g/mL insulin, and 10% FBS) for 4 days, then switched to post differentiation medium containing 10% FBS and 10 *μ*g/mL insulin, and then changed to 10% FBS medium for an additional 2 days to induce differentiation. After differentiation was induced, the 3T3-L1 adipocytes were stained with fresh Oil Red O solution. The 3T3-L1 adipocytes were washed with phosphate buffered saline (PBS) and fixed with 10% formalin. After Oil Red O stain, the cells were photographed using a phase-contrast microscope (Olympus CKX41, Tokyo, Japan) and the lipid droplets were dissolved in isopropanol and quantified by a microplate reader (SYNERGY H1, Microplate reader, BioTek, USA) at 540 nm.

### 2.4. Cell Viability Assay

The 3T3-L1 preadipocytes were plated at 5 × 10^3^cells/well in 96-well plates. AC extract was added to each well at various concentrations (0, 5, 10, 20, 40, 80, 160, and 300 *μ*g/mL) for 24, 48, 72, and 96 h. 20 *μ*L of MTS solution was added 96-well plates, after incubation 30 min, and then optical density at 490 nm was measured using an ELISA reader (SYNERGY H1, Microplate reader, BioTek, USA).

### 2.5. Animal Treatment and Experimental Protocol

Thirty-two 5 week-old male C57BL/6N mice were purchased from Central Lab Animal (SLC, Osaka, Japan), and housed in a temperature- (22 ± 1°C) and humidity (60 ± 5%) controlled room with a cycle of 12-h light and 12-h of darkness for 1 week. After 1 week of rest, mice were discretionally allocated to randomly divided 4 groups (n = 8) as follows: fed a regular diet (RD) group; fed a high-fat diet (HFD, Research Diets, DooYeol Biotech, Seoul, Korea) group; fed a HFD plus AC 100 mg/kg (AC100) group; and fed a HFD plus AC 200 mg/kg (AC200) group (Table 1). Body weights, food and water intakes, and Blood glucose levels of all mice were recorded weekly. After 8 weeks, mice were anesthetized with diethyl ether after an overnight fast. The blood was drawn from the abdominal aorta into a vacuum tube; the epididymal fat tissues were removed, weighed, and frozen with liquid nitrogen. All the animal experimental procedures were in conformity with guidelines and with the approval of the Institutional Animal Care and Use Committees (IACUC) of Hallym University.

### 2.6. Serum Chemistry Analysis

The plasma concentrations of glucose, triglyceride (TG), total cholesterol (TC), and low-density lipoprotein (LDL) cholesterol levels were determined using commercial kits (Thermo Electron Corporation, Vantaa, Finland) and a Thermo Fisher Konelab 20XTi Analyzer (Thermo Electron Corporation, SeoKwang LABOTECH, Seoul, Korea).

### 2.7. Hematoxylin and Eosin Staining

The epididymal adipose tissues were removed and fixed in 10% fresh formalin solution for 24 h. The each group tissue sample was embedded in paraffin wax, sectioned in 5 *μ*m thicknesses, and stained with hematoxylin and eosin (H & E) for microscopic assessment (Axiomager, Zeiss, Germany).

### 2.8. RT-PCR and Real-Time PCR

The total RNA was isolated from the 3T3-L1 adipocytes and epididymal adipose tissue using a Trizol (Invitrogen, Carlshad, CA, USA), and quantified with a NanoDrop-2000 (Thermo Fisher Scientific, Wilmington, DE, USA). The cDNA was generated from 5 *μ*g of total RNA using a Reverse Transcription System kit (Promega, Fitchburg, WI, USA). PCR was performed at 95°C for 30 s followed by annealing for 30 s and then at 72°C for 1 min. The last cycle was followed by a final extension step at 72°C for 10 min. The RT-PCR products were electrophoresed in 0.8% agarose gel under 100 V and were stained with 0.5 *μ*g/mL ethidium bromide. Levels of the reference gene Actin were used to correct for differences in RNA isolation, RNA degradation, and the efficiency of the reverse transcription. Real-time PCR was performed using 1 *μ*L of cDNA in a 20 *μ*L reaction volume with the ABI PRISM 7000 sequence detector system (Applied Biosystems, Foster City, CA, USA). The temperature profile of the reaction was 95°C for 15 min, followed by 30 cycles of denaturation at 95°C for 30 s, and extension at 72°C for 1 min. A relative gene expression quantification method was used to calculate the fold change of mRNA expression according to the comparative threshold cycle method using actin as an endogenous control. Primer sequences are shown in Table 2.

### 2.9. Western Blot Analysis

To detect proteins from whole adipocyte cell lysates, 3T3-L1 cells and epididymal adipose tissue were homogenized in lysis buffer. The protein concentration of the cell lysates was measured using a Bio-Rad protein assay kit (Hercules, CA). The primary antibodies PPAR*γ*, C/EBP*α*, SREBP1, FAS, SCD-1, phosphor-AMPK (p-AMPK), AMPK, phosphor-ACC (p-ACC), ACC, PGC-1*α*, PPAR*α*, ATGL, phosphor-hormone sensitive lipase (p-HSL), and HSL were purchased form Cell Signaling (Danver, USA).

### 2.10. Statistical Analysis

The results are presented as the mean ± SEM, and data were compared using a Student's unpaired* t*-test or one-way analysis of variance (ANOVA), as appropriate. Differences of* p *< 0.05, 0.01, and 0.001 were considered statistically significant.

## 3. Results

### 3.1. Chemical Composition of AC Extract

**Figure 1:**
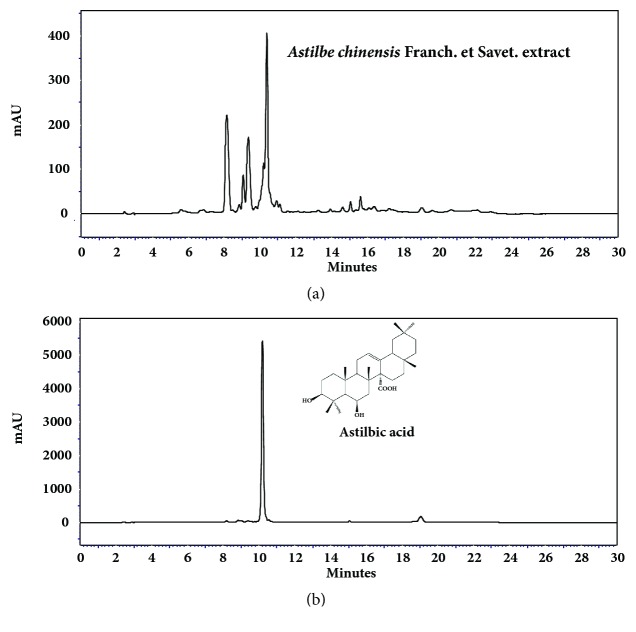
HPLC Chromatograms of* Astilbe chinenesis* Franch. et Savet. extract (a) and the standard astilbic acid (b).

Phytochemical screening of AC extract showed positive results for flavonoids, steroids, and triterpenoids using HPTLC (data not shown). As shown in Figure 1, the major constituents with moderate and high polarity during 0-12 min retention time. The observed triterpenoids were assigned Astilbic acid (3*β*, 6*β*-dihydroxyolean-12-en-27-oic acid) by comparing the retention times with the isolated compounds in HPLC chromatograms and analysis of MS data. The AC extract contained 96.3 ± 0.8 mg/g astilbic acid.

### 3.2. Effect of AC Extract on Adipocyte Differentiation

**Figure 2:**
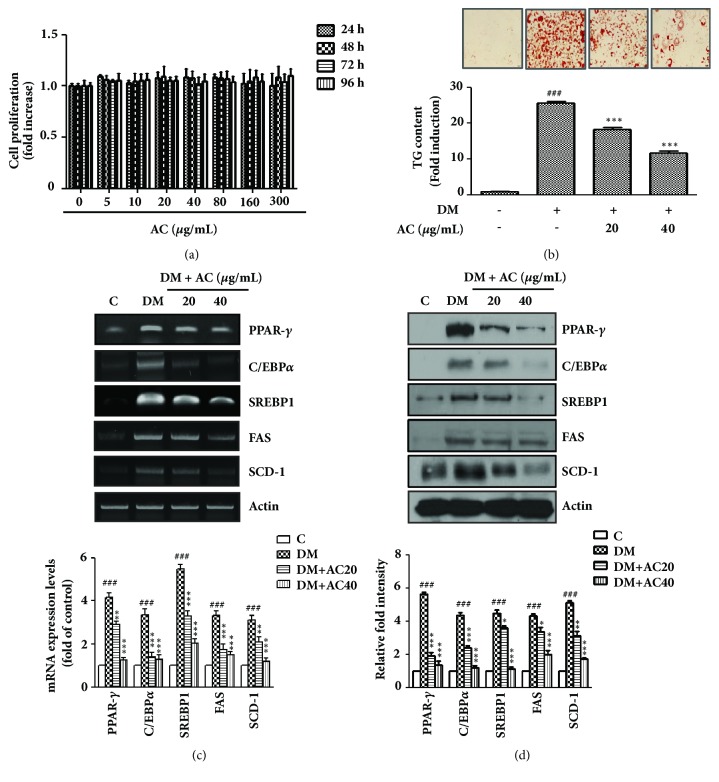
Effects of the* Astilbe chinensis *Franch. et Savat. (AC) extract on inhibiting 3T3-L1 adipocyte differentiation. (a) The 3T3-L1 cell proliferation was measured by MTS assay after 24 h, 48 h, 72 h, and 96 h treatment of AC extract at various concentrations. (b) The 3T3-L1 cells differentiated with differentiation medium in the absence or presence of AC extract for 8 days. Intracellular lipids were stained with Oil Red O, and triglyceride content was quantified by spectrometry at 540 nm. (c) mRNA levels of PPAR-*γ*, C/EBP*α*, SREBP1, FAS, and SCD-1, as determined by reverse transcription PCR (upper panels) and real-time PCR (lower panels), and (d) the protein expression levels of PPAR-*γ*, C/EBP*α*, SREBP1, FAS, and SCD-1 were examined by western blot analyses. Data are presented as the mean ± SEM of three independent experiments, each performed in triplicate. ^###^*p*< 0.001* vs* untreated cells; ^*∗*^*p*< 0.05, ^*∗∗*^*p*< 0.01 and ^*∗∗∗*^*p*< 0.001* vs* differentiated cells (DM).

We first performed an MTS assay to assess the effect of AC extract on 3T3-L1 cell viability. As shown in Figure 1(a), the treatment of 5, 10, 20, 40, 80, 160, and 300 *μ*g/mL of AC extract for 24, 48, 72, and 96 h resulted in no significant effects on cell viability. Next, to elucidate the effect of AC extract on the differentiation of preadipocytes into adipocytes, 3T3-L1 cells were treated with the indicated concentrations of AC extract and lipid accumulation was examined by using Oil Red O staining. As shown in Figure 2(b), AC extract reduced lipid droplet size compared with cells grown in differentiated media. Differentiation of 3T3-L1 adipocytes was inhibited by treatment with AC extract, and triglyceride content in 3T3-L1 adipocytes significantly decreased in AC extract-treated cells at 20 and 40 *μ*g/mL. RT-PCR and western blotting analyses were used to quantify the expression of adipogenesis markers, such as peroxisome proliferator-activated receptor-*γ* (PPAR-*γ*), CCAAT/enhancer binding protein *α* (C/EBP*α*), and sterol regulatory element-binding protein-1 (SREBP-1) in 3T3-L1 adipocytes. However, AC extract caused significant downregulation of the expression of PPAR-*γ*, C/EBP*α*, and SREBP1 and its target molecules, fatty acid synthase (FAS), and stearoyl-CoA desaturase-1 (SCD-1), in comparison with differentiated cells.

### 3.3. Activation of AMPK Pathway by AC Extract in 3T3-L1 Adipocytes

**Figure 3:**
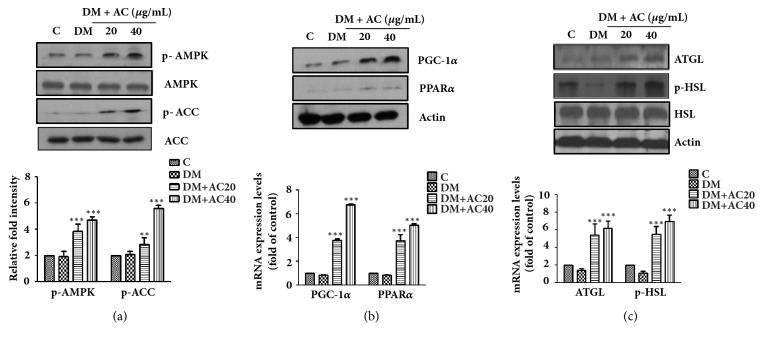
The effect of* Astilbe chinensis *Franch. et Savat. (AC) extract on gene expression in 3T3-L1 adipocytes. (a) Expression of AMPK, ACC phosphorylation, (b) PGC-1*α*, PPAR*α* and (c) ATGL, p-HSL mRNA and protein activated in the 3T3-L1 adipocytes and normalized by western blot (upper) and real-time PCR (lower). Data are presented as the mean ± SEM of three independent experiments, each performed in triplicate. ^*∗*^*p*< 0.05, ^*∗∗*^*p*< 0.01, and ^*∗∗∗*^*p*< 0.001 compared with differentiated cells (DM).

AMPK is an important cellular energy metabolism sensor with a key role in the regulation of adipogenesis and lipolysis [11, 12]. We next examined whether AC extract stimulated the phosphorylation of AMPK in 3T3-L1 adipocytes. As shown in Figure 3(a), treatment with AC extract resulted in a significant dose-dependent activation of AMPK and ACC. Furthermore, the protein and mRNA expression of PGC-1*α*, PPAR*α*, ATGL, and HSL, which are critical regulators of mitochondrial biogenesis and lipolysis, were significantly increased by AC extract treatment at 40 mg/mL (Figures 3(b) and 3(c)). These data indicated that AC extract treatment inhibited adipogenesis and increased lipolysis mediated by AMPK activation.

### 3.4. Effects of AC Extract on Body Weight and Biomarkers in High-Fat Diet Induced Obese Mice

To assess whether AC extract caused an anti-adipogenic and lipolytic effect in vivo, we used an HFD-induced obese model. The effects of 100 and 200 mg/kg AC extract on body weight and metabolic parameters in high-fat-diet-induced obese mice treated for 8 weeks are shown in Table 3. There was no significant difference in the initial body weight and the food intake between the RD and HFD groups. However, the body weight gains in AC extract-treated mice were significantly smaller than those in the HFD group, after 8 weeks on the experimental diet (55% decrease in the AC100 group and 61% decrease in the AC200 group). At the end of the treatment period, the changes in plasma glucose, insulin, and lipid levels were also examined. The plasma glucose and insulin levels were higher in the HFD group than in the RD group, but AC extract treatment significantly reduced the plasma glucose and insulin levels in a concentration-dependent manner. The AC100 and AC200 groups showed a significant decrease in the levels of TG, TC, and LDL-cholesterol (22%, 56%, and 40% inhibition in the AC200 group, respectively).

### 3.5. Effects of AC Extract on Epididymal Adipose Tissue

**Figure 4:**
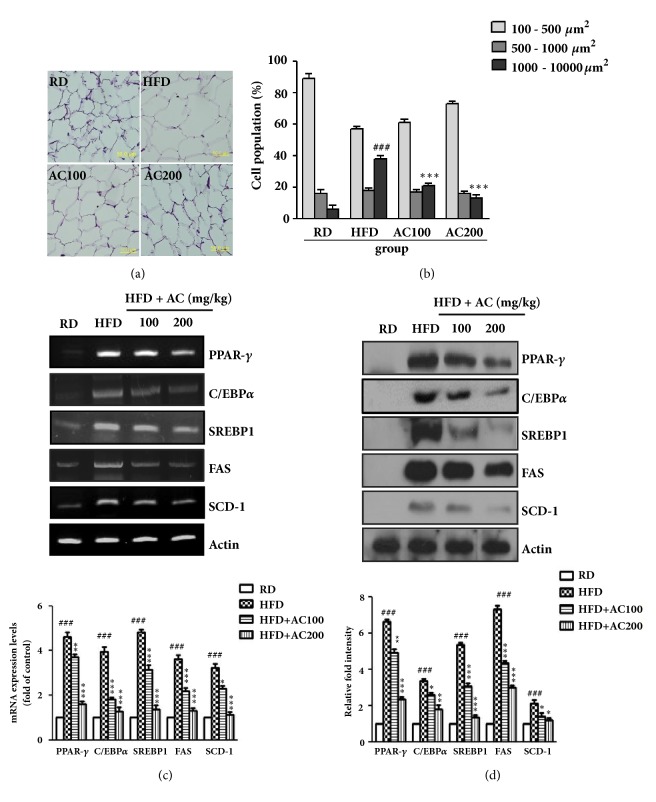
Effects of* Astilbe chinensis *Franch. et Savat. (AC) extract on epididymal fat size in HFD induced obese mice. (a) The H & E stained epididymal fat tissue from HFD induced obese mice treated with RD or AC (100 and 200 mg/kg), and (b) quantification of epididymal fat tissues size were calculated. The (c) mRNA levels of PPAR-*γ*, C/EBP*α*, SREBP1, FAS, and SCD-1, as determined by reverse transcription PCR (upper panels) and real-time PCR (lower panels), and (d) the protein levels of PPAR-*γ*, C/EBP*α*, SREBP1, FAS, and SCD-1 were examined by western blot analyses, respectively. ^###^*p*< 0.001* vs* RD mice; ^*∗*^*p*< 0.05, ^*∗∗*^*p*< 0.01, and ^*∗∗∗*^*p*< 0.001 compared with HFD mice.

To elucidate the weight-reducing effect of AC extract in HFD-induced obese mice, the volume of adipose tissues was measured by using H & E staining. The epididymal adipocyte size was significantly larger in the HFD group than in the RD group, whereas the adipocyte size was smaller in the AC extract-treated group than the untreated HFD-induced obese mice (Figures 4(a) and 4(b)). To evaluate the AC extract-induced reduction in adipocyte size, we measured the gene and protein expression in the epididymal adipose tissue of HFD-induced obese mice. The gene expression of PPAR-*γ*, C/EBP*α*, SREBP1, FAS, and SCD-1 was dose-dependently reduced in AC extract-treated mice compared with HFD mice (Figures 4(c) and 4(d)).

### 3.6. AC Extract Regulated Gene Expression of Lipid Metabolism in Epididymal Fat Tissue

**Figure 5:**
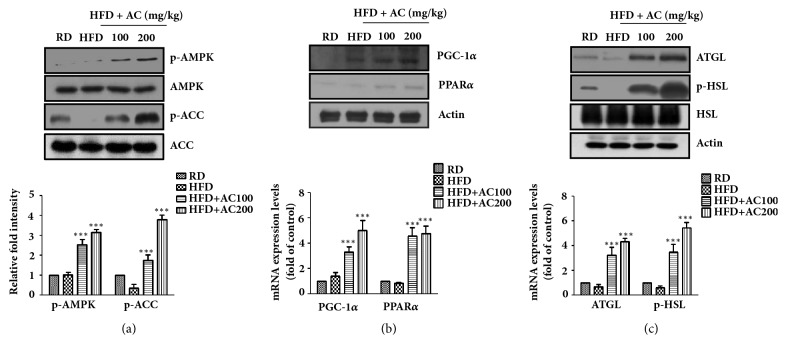
Effects of* Astilbe chinensis *Franch. et Savat. (AC) extract on AMPK and ACC phosphorylation (a), PGC-1*α*, PPAR*α* (b) and ATGL, p-HSL (c) gene expression in the epididymal fat, and determined by western blot (upper) and real-time PCR (lower). ^*∗∗∗*^*p*< 0.001 compared with HFD mice.

The current literature indicates that adipose triglyceride lipase (ATGL) and hormone-sensitive lipase (HSL) are the major lipases for approximately 95% of lipase activity in white adipose tissue [13]. Thus, we examined the effects of AC extract on the AMPK pathways in epididymal fat tissue. In contrast, pAMPK, pACC, PGC-1*α*, PPAR-*α*, ATGL, and HSL were markedly induced in AC extract-treated mice, as shown in Figure 5.

## 4. Discussion

Obesity is considered a major cause of several metabolic diseases, including type 2 diabetes mellitus, hypertension, dyslipidemia, and cardiovascular disease [14, 15]. Although many scientists are actively developing therapies for obesity, natural products suitable for the treatment of obesity have been frequently suggested as an alternative strategy owing to the severe adverse effects induced by antiobesity drugs [16]. In this study, we investigated the antidifferentiation effect of AC extract on 3T3-L1 preadipocytes and explored the antiobesity activity of AC extract in HFD-induced obese C57BL/6N mice.

It is well known that adipogenesis is a differentiation process associated with the expansion of adipose tissue and induces obesity [17, 18]. During adipocyte differentiation, adipocyte markers, such as PPAR-*γ*, C/EBP*α*, and SREBP1, are considered key regulators of adipogenesis [19, 20]. In the present study, AC extract significantly decreased the mRNA and protein expression of the adipogenic transcription factor genes PPAR-*γ*, C/EBP*α*, and SREBP1 (Figure 2) and its target molecules FAS and SCD-1 in differentiated 3T3-L1 cells and the epididymal fat tissue of HFD-induced obese mice. These results suggest that AC extracts may prevent weight gain in HFD-induced obese C57BL/6N mice.

Recent evidence has shown that basic cellular metabolic pathways play a major role in the regulation of whole-body energy homeostasis [21]. Among them, a master sensor and regulator of energy homeostasis, the AMPK pathway, has been examined at the cellular level [22–24]. AMPK activates the catabolic processes that provide alternative routes for ATP generation, glucose uptake, glycolysis, fatty acid oxidation, and mitochondrial biogenesis. The PPAR-*γ* coactivator-1*α* (PGC-1*α*) and PPAR-*α* are critical regulators of mitochondrial biogenesis and energy expenditure induced by AMPK activation [25, 26]. However, as shown in Figures 3 and 5, AC extract stimulated the phosphorylation of AMPK, PGC-1*α*, and PPAR-*α* in a dose-dependent manner. These results indicated that AC extracts induced the activation of the AMPK-mediated gene expression increase in PGC-1*α* and PPAR*α*, which drove lipid metabolism toward energy dissipation instead of storage.

**Figure 6:**
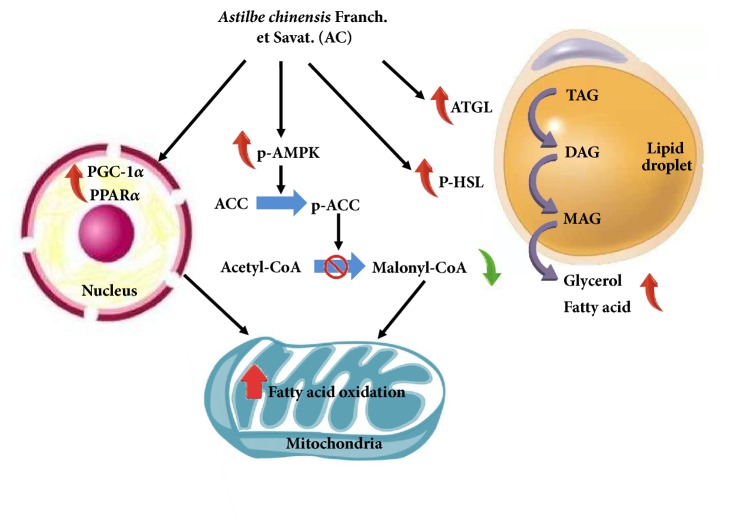
Proposed antiobesity mechanisms of* Astilbechinensis *Franch. et Savat.

ATGL and HSL are major regulators of lipolysis in the white adipose tissue of rodents [27, 28]. It is well known that ATGL initiates lipolysis through the cleavage of the first fatty acids (FAs) from triacylglycerols (TAGs), which is followed by the action of HSL and MAGL on diacylglycerols (DAGs) and monoacylglycerols. However, recent research has shown that AMPK, via a cAMP-dependent mechanism, is required for the activation of lipolysis in 3T3-L1 adipocytes [29] and stimulates lipolysis in 3T3-L1 adipocytes through the phosphorylation of HSL [30]. Thus, the orchestrated activation of AMPK, ATGL, and HSL appears to be a requirement for the occurrence of complete lipolysis in adipocytes. In the present study, AC extract was found to stimulate lipolysis in HFD-induced obese mice and 3T3-L1 adipocytes through a direct increase in the expression of ATGL and the phosphorylation of HSL, AMPK, and ACC (Figure 6).

In conclusion, we demonstrated that supplementation with AC extract significantly suppressed adipocyte differentiation in 3T3-L1 cells, as well as body weight gain, feed efficiency levels, adipose tissue size, and the serum levels of glucose, insulin, TG, and TC in HFD-induced obese C57BL/6N mice. Furthermore, AC extract altered the expression of many genes that are related to adipocyte differentiation and lipolysis. In summary, these results have provided molecular-level information to permit further investigation of the mechanisms through which AC extract moderated lipid metabolism. Further investigations are required to identify the responsible compounds in AC and their mechanisms of action.

## Figures and Tables

**Table 1:** Composition of the experimental diets.

**Component (g/kg)**	**RD**	**HFD**
Casein	210	265
L-cystine	3	4
Corn starch	280	-
Maltodextrin	50	160
Sucrose	325	90
Lard	20	310
Soybean oil	20	30
Cellulose	37.15	65.5
Mineral mixture	35	48
Vitamin mixture	15	21
Calcium phosphate, dibasic	2	3.4
Choline bitartrate	2.75	3
Yellow food color	0.1	-
Blue food color	-	0.1
Total (g)	1000	1000
Protein (% of kcal)	20.1	18.3
Carbohydrate (% of kcal)	69.8	21.4
Fat (% of kcal)	10.2	60.3
Energy (kcal/g)	3.7	5.1

**Table 2:** Primer sequence.

**Gene**	**Primer Sequence (**5′** → **3′**)**		**Annealing**
	**Forward primer**	**Reverse primer**	**temp (**°**C)**
PPAR-*γ*	TTTTCAAGGGTGCCAGTTTC	AATCCTTGGCCCTCTGAGAT	58
C/EBP*α*	AGACATCAGCGCCTACATCG	TGTAGGTGCATGGTGGTCTG	58
SREBP1	GCGCTACCGGTCTTCTATCA	TGCTGCCAAAAGACAAGGG	58
FAS	GATCCTGGAACGAGAACAC	AGACTGTGGAACACGGTGGT	50
SCD-1	CGAGGGTTGGTTGTTGATCTGT	ATAGCACTGTTGGCCCTGGA	56
PGC-1*α*	CACCAAACCCACAGAAAACAG	GGGTCAGAGGAAGAGATAAAGTTG	60
PPAR*α*	ATGAAGAGGGCTGAGCGTAGGTAA	TGCCGTTGTCTGTCACTGTCTGAA	57
ATGL	ACCAACACCAGCATCCAGTT	TTTGCACATCTCTCGGAGGA	56
HSL	TTCGAGGGTGATGAAGGACT	ACTCTGGGTCTATGGCGAAT	58
Actin	GTCGTACCACTGGCATTGTG	GCCATCTCCTGCTCAAAGTC	60

**Table 3:** Effects of *Astilbe chinensis *Franch.et Savat. extract on body weight and biomarkers in mice fed a high-fat diet.

**Groups**	**RD **	**HFD**	**AC 100**	**AC 200**
Initial body weight (g)	21.2 ± 1.5^NS^	21.7 ± 1.2^NS^	21.8 ± 1.3^NS^	21.7 ± 1.4^NS^
Final body weight (g)	29.8 ± 2.3^a^	43.5 ± 2.4^c^	31.8 ± 1.9^ab^	30.2 ± 2.3^ab^
Body weight gain (g)	8.5 ± 1.9^a^	21.6 ± 1.5^c^	9.8 ± 1.3^ab^	8.4 ± 1.6^ab^
Food intake (g/day)	3.13 ± 0.2^NS^	3.26 ± 0.3^NS^	3.31 ± 0.2^NS^	3.28 ± 0.2^NS^
Feed efficiency	0.72 ± 0.1^a^	3.66 ± 0.2^c^	1.79 ± 0.1^b^	1.33 ± 0.2^ab^
Plasma glucose (mM)	5.3 ± 0.7^a^	8.6 ± 0.8^c^	6.1 ± 1.3^b^	6.0 ± 0.4^b^
Plasma insulin (*μ*U/mL)	60.1 ± 6.8^a^	163.6 ± 13.7^c^	87.3 ± 7.7^b^	74.3 ± 11.3^ab^
TG (mg/mL)	48.7 ± 6.9^a^	88.3 ± 9.9^c^	42.3 ± 7.0^a^	68.5 ± 22.6^b^
TC (mg/mL)	50.8 ± 4.0^a^	116.1 ± 5.6^b^	58.4 ± 6.5^a^	51.1 ± 11.2^a^
LDL-cholesterol (mg/mL)	13.3 ± 2.3^a^	32.3 ± 3.2^c^	21.1 ± 1.8^b^	19.3 ± 2.1^b^

Results are presented as mean ± SEM (n = 8). Values within a row with different letters are significantly different from each other (*p*< 0.05). RD: regular diet group; HFD, high-fat diet group; AC 100, HFD + *Astilbe chinensis *Franch. et Savat. (AC) extract 100 mg/kg treatment; AC 200, HFD + *Astilbe chinensis *Franch. et Savat. (AC) extract 200 mg/kg treatment; TG: triacylglycerol; TC: total cholesterol; LDL: low-density lipoprotein; ^NS^: not significant.

## Data Availability

The data used to support the findings of this study are available from the corresponding author upon request.
